# Maternal Decision-Making Input and Health-Seeking Behaviors Between Pregnancy and the Child’s Second Birthday: A Cross-Sectional Study in Nepal

**DOI:** 10.1007/s10995-020-02961-z

**Published:** 2020-06-16

**Authors:** Zhongjie Zhang, Kenda Cunningham, Ramesh Prasad Adhikari, Subash Yogi, Shraddha Manandhar, Pooja Pandey Rana, Anne Paxton

**Affiliations:** 1 Department of Epidemiology, Mailman School of Public Health, Columbia University, New York, NY USA; 2 Helen Keller International, Kathmandu, Nepal; 3 CARE, Kathmandu, Nepal

**Keywords:** Decision-making, Health-seeking behaviors, Maternal, newborn and child health (MNCH) services, First 1000 days, Nepal

## Abstract

**Objectives:**

Maternal health-seeking behaviors are critical to improving maternal and child health in low-income countries. This study investigates associations between maternal decision-making input and their health-seeking behaviors in the first 1000-day period between pregnancy and a child’s second birthday in Nepal.

**Methods:**

We used data from a cross-sectional survey conducted in 2018 in 16 districts of Nepal. Among the 3648 households surveyed, 1910 mothers of a child 0 to 24 months with complete data were included for analyses. Logistic regression was used to examine associations between decision-making input and the utilization of antenatal, delivery and postnatal care services, and attendance at health mothers’ group (HMG) meetings. We also used negative binomial regression to assess the relationship between her decision-making input and participation in growth monitoring and promotion (GMP) in the 6 months prior to the survey. For each relationship examined, we adjusted for clustering, as well as potentially confounding factors at individual and household levels.

**Results:**

After adjusting for confounders, maternal decision-making input had a small but positive and significant association with receiving at least 4 antenatal care visits (OR = 1.09, 95% CI 1.02, 1.17), attendance at GMP in the 6 months prior to the survey (IRR = 1.02, 95% CI 1.00, 1.04), and HMG attendance (OR = 1.10, 95% CI 1.03, 1.17), but not with receiving at least 3 postnatal care visits or delivering in a health institution.

**Conclusions for Practice:**

Our findings indicated that empowering women and mothers in household decision-making might warrant greater attention when developing future policies and programs in Nepal.

## Significance

Previous research reports mixed results on the relationship between maternal decision-making and their health seeking behaviors in low-and middle-income countries (LMICs). This study shows that maternal decision-making input is positively associated with the utilization of a variety of health services during the first 1000-day period in Nepal. Although other factors may be stronger determinants, the findings of this study suggest shifting social norms so that women and mothers are empowered to make household decisions should be considered when developing future policies and programs to make further progress in improving maternal and child health in Nepal and other LMICs.

## Introduction

Maternal health-seeking behaviors in the first 1000 days between the start of pregnancy and a child’s second birthday are of critical importance to promote the health and nutritional well-being of both mothers and children. Global evidence has suggested that adequate use of antenatal, perinatal and postpartum care is effective in improving birth outcomes and averting maternal deaths (Bhutta et al. 2014; Campbell and Graham 2006). Maintaining good nutritional status during pregnancy and receiving quality care after childbirth also play an essential role in enhancing child survival and intellectual development (Bryce et al. 2008). Moreover, appropriate early development in the first 1000-day period lays the foundation for the long-term benefits the child enjoys in regards to health, employment and attaining his or her full potential (Hurt et al. 2018).

Findings from previous Nepal Demographic and Health Surveys (DHS) suggest an increased utilization of maternal, newborn, and child health (MNCH) services and nutrition interventions (Ministry of Health and Population (MOHP) Nepal, New ERA, & ICF International Inc. 2012; Ministry of Health and Population (MOHP) Nepal, New ERA, & Macro International Inc. 2007), but the 2016 Nepal DHS shows that the utilization of health services among mothers aged 15–49 remained relatively low, with only 58% giving birth with the assistance of skilled providers and 57% receiving a postnatal check within 2 days after delivery (Ministry of Health Nepal, New ERA, & ICF 2017). An integrated nutrition program, known as *Suaahara II* (SII), aims to improve the nutritional status of women and children within the first 1000-day period throughout 42 of Nepal’s 77 districts, in part by increasing both supply and quality as well as demand for a host of MNCH services provided at health facilities and by community-based female community heath volunteers (FCHVs).

A growing body of research has explored potential factors impeding women and mothers from accessing adequate health services in low-and middle-income countries (LMICs). Long distances to health clinics, poor public transportation, and lack of control over resources are some of the commonly reported barriers (Banke-Thomas et al. 2017; Bohren et al. 2014). The role of maternal decision-making in her health-seeking behaviors has drawn increasing research interest globally. This particular relationship also deserves careful examination in South Asia, where socio-cultural norms have long prevented women from making their own strategic life choices (Bloom et al. 2001; Mistry et al. 2009), and young mothers’ low status in the household is often considered as an important risk factor for multiple adverse health outcomes they might experience (Gill and Stewart 2011). The most recent pooled analysis drawing on DHS data from 63 LMICs reported positive associations between women’s autonomy in decision-making and the utilization of antenatal care (ANC) services and facility delivery (Sripad et al. 2019). However, studies conducted in Nepal and secondary analyses using Nepal DHS data that explored similar associations identified mixed results, with the presence of both positive relationships and non-significant findings (Furuta and Salway 2006; Joshi et al. 2014; Shah et al. 2015; Situ and Neupane 2016). In addition, the main focus of the existing research is primarily on the use of ANC and institutional delivery, and none of them to date has comprehensively examined the role of maternal decision-making in the utilization of a wide variety of health services across the first 1000-day period.

To fill the research gap, this study aims to investigate the associations between maternal decision-making input and their health-seeking behaviors during pregnancy and lactation including for ANC, institutional delivery, postnatal care (PNC), growth monitoring and promotion (GMP) and health mothers’ group (HMG) meetings in Nepal.

## Methods

### Study Population and Sampling

We used data from SII’s 2018 cross-sectional monitoring dataset, collected by New ERA, a local survey firm in Nepal. Multistage cluster sampling was used to select a survey population representative of the overall SII intervention areas, with probability proportion to size (PPS) used in the first four sampling stages, resulting in 16 districts, 32 municipalities (2 per district), 96 new wards (3 per municipality) and 192 old wards (2 per new ward). In each old ward, households with a child under 5 years, the final sampling unit, were randomly selected from a complete listing. A total of 3648 households (19 per old ward) was eventually enrolled in this survey. The primary survey respondents were mothers of children under 5 years and a household head, with preference given to her spouse or another major adult male decision-maker.

Given focus on the first 1000-day period, we restricted our study population to households with a child under 2 years who completed the survey (n = 1910). For analyses with the attendance of GMP and HMG meetings, the outcomes measured within the 6 months prior to the survey, we limited them to households with a child at least 6 months (N = 1460).

Written inform consent was obtained from each respondent before the interview. Ethical approval was obtained from the Nepal Health Research Council (NHRC).

### Measurement of Primary Outcomes, Primary Exposures, and Other Predicator Variables

Primary study outcomes include: ANC, institutional delivery, PNC, GMP and HMG, which were all measured by maternal recall. ANC was constructed as a binary variable and was coded as 1 if the mother had at least 4 ANC visits during pregnancy, the minimum frequency recommended by the World Health Organization (WHO) and incorporated into Nepal’s National Safe Motherhood Programme (Ministry of Health and Population (MOHP) Nepal 2009; Villar 2001). Institutional delivery was measured as to whether she gave birth at any public or private health facility. PNC was classified, based on Nepal’s protocol, that mothers should have at least 3 PNC visits within the first week after delivery (Kearns et al. 2014). Given that GMP guidelines in Nepal state that a child should be weighed once per month and HMG meetings are a monthly community activity led by FCHVs to disseminate critical health and nutrition information and provide support to mothers during the postpartum period (Ministry of Local Development of Nepal & UNICEF 2007), GMP and HMG attendance frequency in the 6 months prior to the survey were measured as count variables. HMG attendance did not follow a Poisson distribution; thus, we converted this into a binary variable of none versus at least 1 attendance at HMG meetings. For all 5 outcome variables, anyone who answered “don’t know” (4 respondents for ANC and 3 for GMP) was coded as not using the health service.

The primary exposure of maternal decision-making input was measured using their responses to survey questions about the participation in decisions on 13 household activities (Table 1). These 13 survey questions were adapted from the decision-making section of the validated and widely-used Women Empowerment in Agriculture Index (WEAI), and modified to be specific to SII thematic intervention areas (Malapit et al. 2019). For each question, response options included: (1) little to no input, (2) input into some decisions, (3) input into most or all decisions, and 97) N/A: decisions are not made. We created binary variables to define subjects as having adequate decision-making input if they reported input into most or all decisions (coded as 1). The sum of the 13 binary indicators became the composite score to measure mothers’ household decision-making input; its Cronbach coefficient of 0.75 indicated acceptable internal consistency.

**Table 1:** Operationalization of decision-making variable

Variable	Decision-making question	Response options	Operationalization
Composite score of decision-making input	How much input did you have in making decisions about: (a) horticultural gardens or high value crop farming (b) poultry or chicken (c) non-farm economic activities (d) wage and salary employment (e) large occasional household purchase (f) routine household purchase (g) use of family planning methods (h) your health care (i) food consumption (j) children’s health care (k) feeding children (l) purchase and use of water treatment (m) going out of the house to engage in the community	(1) = Little to no input (2) = Input into some decisions (3) = Input into most or all decisions (97) = N/A: decisions not made	For each survey question, providing a response of “(3) input into most or all decisions" is considered as having adequate input into the respective decision-making domain, and every decision-making variable is then dichotomized: Coded 1 if response was (3) Coded 0 if response was not (3) We aggregated these 13 equally weighted individual binary variables to compute a composite score measuring maternal household decision-making input, with a possible range from 0 to 13

The following socio-demographic characteristics were included in the study as potentially confounding factors: child age (completed months) and sex (boy/girl); maternal age (completed years), education (years of formal schooling), parity (total number of times a mother gave birth), and whether living with mother-in-law (yes/no); and household caste/ethnicity, equity quintile, urban/rural residency, and agro-ecological zone of residence (hills, mountains, and *terai* (lowlands). Caste/ethnicity was constructed as 3 categories: lower (i.e. Dalit, Muslim, disadvantaged Janjati), relatively advantaged (i.e. Newar, Gurung/Thakali, non-Dalit *terai* caste, others) and upper (Brahmin and Chhetri) (Bennett et al. 2006). Using the Equity Tool (The EquityTool 2018), we created equity quintiles (lowest, second lowest, middle, second highest, and highest) reflecting a household’s socio-economic well-being relative to the wealth status of the national population measured by the 2016 Nepal DHS.

### Statistical Analysis

Descriptive analyses of socio-economic and demographic characteristics, maternal decision-making input and health-seeking behaviors in our study population were done. Regression models were used to assess associations between maternal decision-making input and the five outcomes of interest. Logistic regression models were used for binary outcomes including ANC, institutional delivery, PNC and HMG attendance. Negative binomial regression modelling was used for models with GMP as the outcome, because GMP had a variation larger than its mean (over-dispersed). Crude and adjusted odds ratios and incidence rate ratios, along with their respective 95% confidence intervals, were obtained.

All data were analyzed using Stata SE 14.0 (Stata Corporation, College Station, TX). Command option “cluster” was employed to account for the effect of cluster sampling on the standard error of each parameter estimate when constructing regression models.

## Results

Table 2 describes socio-economic and demographic characteristics of the 1910 mothers in the study and the study’s primary explanatory and outcome variables, relating to maternal decision-making input and health-seeking behaviors within the 1000-day period. The mothers’ mean age was 24 years and they had, on average, 7 years of schooling. They had given birth to an average of 2 children and most (59.3%) were living with their mother-in-law. More than half (51.9%) of the mothers were from lower caste-ethnic groups, although 40.1% of them were from upper caste groups. Nearly 70% of the households were categorized as being in the middle equity quintile or lower. More than half (57.1%) were residing in the hills. Among these children 0–2 years, their mean age was 11.6 months and slightly more than half (52.9%) were boys.

**Table 2:** Socio-economic and demographic characteristics, maternal decision-making and health seeking behaviors among mothers in Nepal (N = 1910)

Variables	Mean (SD)/%
Maternal characteristics	
Age (years) (range: 15–46)	24.4 (4.8)
Education (years) (range: 0–17)	7.1 (4.0)
Parity (range: 1–10)	2.0 (1.3)
Household characteristics	
Caste/ethnicity	
Lower caste/ethnic groups	51.9%
Relatively advantaged caste/ethnic groups	8.0%
Upper caste/ethnic groups	40.1%
Equity quintile	
Lowest quintile	18.0%
Second lowest quintile	24.7%
Middle quintile	25.5%
Second highest quintile	23.8%
Highest quintile	8.0%
Residency (rural)	50.2%
Agro-ecological zone of residence	
Mountain	12.4%
Hill	57.1%
*Terai*	30.5%
Living with mother-in-law	59.3%
Child characteristics	
Age (months) (range: 0–23)	11.6 (6.9)
Sex (boys)	52.9%
Primary exposure: maternal decision-making input	
Composite score of decision-making input (range: 0–12)	5.8 (2.5)
Primary outcome: maternal health-seeking behaviors	
Among mothers with a child 0–24 months of age (N = 1910)	
Having at least 4 ANC during pregnancy	85.5%
Having institutional delivery	77.3%
Having at least 3 PNC in the first week after delivery	33.1%
Among mothers with a child 6–24 months of age (N = 1460)	
Number of GMP in the 6 months prior to the survey	2.3 (1.9)
Number of HMG attendance in the 6 months prior to the survey	0.9 (1.9)
No attendance at HMG meetings	77.5%
At least 1 attendance at HMG meetings	22.5%

*ANC* antenatal care, *PNC* postnatal care, *GMP* growth monitoring and promotion, *HMG* health mothers’ group

The mean score of maternal decision-making input was 5.8 points on a scale of 0 to 13. Almost all mothers (85.7%) made at least 4 ANC visits during pregnancy, 77.3% gave birth in a health institution, and 33.1% obtained PNC at least 3 times in the first week after delivery. Among mothers with a child 6–24 months of age, they made, on average, 2.3 of possible 6 visits to 6 GMP in the 6 months prior to the survey, and only 22.5% of them attended at least 1 HMG meeting.

Table 3 presents the results of unadjusted associations between maternal decision-making input and health-seeking behaviors in the first 1000-day period. Among mothers with a child 6–24 months of age, every one-point increase in the composite score of maternal decision-making input was associated with an 11.1% increase in the odds of having HMG attendance (OR = 1.11, 95% CI 1.05, 1.18). However, we did not find significant associations between maternal decision-making input and the utilization of other health services studied.

**Table 3:** Unadjusted associations between maternal decision-making input and their health seeking behaviors in the first 1000-day period between pregnancy and a child’s second birthday among mothers in Nepal

	Among mothers with a child aged 0–24 months (N = 1910)			Among mothers with a child aged 6–24 months (N = 1460)	
	At least 4 ANC	Institutional delivery	At least 3 PNC in the first week after delivery	Number of GMP in the 6 months prior to the survey	HMG attendance
	OR (95% CI)	OR (95% CI)	OR (95% CI)	IRR (95% CI)	OR (95% CI)
Composite score of decision-making input	1.04 (0.98, 1.10)	0.97 (0.92, 1.02)	1.02 (0.98, 1.06)	1.00 (0.99, 1.02)	1.11** (1.05, 1.18)

*ANC* antenatal care, *PNC* postnatal care, *GMP* growth monitoring and promotion, *HMG* health mothers’ group, *OR* odds ratio, *IRR* incidence rate ratio

*P < 0.05

**P < 0.01

In Table 4, the results of associations between maternal decision-making input and all 5 health-seeking behaviors in the 1000-day period, adjusted for individual and household characteristics mentioned above and clustering, are shown. \The odds of having at least 4 ANC during pregnancy was estimated to increase by 9.1% (OR = 1.09, 95% CI 1.02, 1.17) on average for every one-point increase in the composite score reflecting maternal decision-making input. Among mothers with a child aged 6–24 months, every one-point increase was also associated with a 2.1% higher incidence rate of attending GMP (IRR = 1.02, 95% CI 1.00, 1.04) and a 10.0% increase in the odds of having at least 1 attendance of monthly HMG meetings (OR = 1.10, 95% CI 1.03, 1.17). On the other hand, no significant associations were found between maternal decision-making input and institutional delivery or having at least 3 PNC in the first week after delivery. Among socio-economic and demographic characteristics, maternal education was positively associated with almost all health-seeking behaviors studied. For every one-year increase in the education a mother received, the adjusted odds of having at least 4 ANC, institutional delivery, at least 3 PNC in the first week after delivery and the adjusted incidence rate of attending monthly GMP were expected to increase by 3% to 9%. In addition, some household characteristics, such as upper caste/ethnicity, highest equity quintile, and rural residency, also showed positive relationships with improved utilization of maternal health services.

**Table 4:** Adjusted associations between maternal decision-making input and their health seeking behaviors in the first 1000-day period between pregnancy and a child’s second birthday among mothers in Nepal

	Among mothers with a child aged 0–24 months (N = 1910)			Among mothers with a child aged 6–24 months (N = 1460)	
	At least 4 ANC	Institutional delivery	At least 3 PNC in the first week after delivery	Number of GMP in the 6 months prior to the survey	HMG attendance
	OR (95% CI)	OR (95% CI)	OR (95% CI)	IRR (95% CI)	OR (95% CI)
Composite score of decision-making input	1.09* (1.02, 1.17)	1.04 (0.98, 1.09)	1.05 (1.00, 1.10)	1.02* (1.00, 1.04)	1.10** (1.03, 1.17)
Maternal age	1.00 (0.96, 1.03)	1.02 (0.99, 1.05)	1.01 (0.98, 1.04)	1.01 (1.00, 1.02)	1.03 (1.00, 1.07)
Maternal education	1.09** (1.05, 1.14)	1.09** (1.04, 1.14)	1.04** (1.01, 1.07)	1.03** (1.01, 1.04)	1.05 (1.00, 1.10)
Maternal parity	0.82** (0.73, 0.93)	0.78** (0.68, 0.88)	0.92 (0.81, 1.04)	0.91** (0.86, 0.96)	1.15 (0.98, 1.33)
Household caste/ethnicity					
Lower caste/ethnic groups	1.00 (ref)	1.00 (ref)	1.00 (ref)	1.00 (ref)	1.00 (ref)
Relatively advantaged caste/ethnic groups	0.69 (0.40, 1.19)	0.91 (0.60, 1.39)	0.82 (0.54, 1.26)	0.71** (0.58, 0.88)	0.37* (0.17, 0.79)
Upper caste/ethnic groups	1.38 (0.93, 2.03)	1.60* (1.12, 2.28)	1.15 (0.90, 1.47)	1.04 (0.93, 1.16)	1.66** (1.16, 2.38)
Household equity quintile					
Lowest quintile	1.00 (ref)	1.00 (ref)	1.00 (ref)	1.00 (ref)	1.00 (ref)
Second lowest quintile	1.15 (0.74, 1.80)	0.74 (0.54, 1.01)	1.26 (0.87, 1.82)	0.97 (0.84, 1.12)	0.87 (0.56, 1.36)
Middle quintile	1.93** (1.21, 3.06)	1.37 (0.95, 1.99)	1.46 (0.96, 2.21)	0.87 (0.75, 1.01)	0.80 (0.48, 1.35)
Second highest quintile	3.15** (1.90, 5.21)	2.07** (1.40, 3.07)	1.53 (0.99, 2.36)	0.86 (0.73, 1.01)	0.88 (0.50, 1.56)
Highest quintile	2.25* (1.16, 4.37)	2.65** (1.34, 5.24)	2.23** (1.25, 3.99)	0.91 (0.73, 1.13)	0.28** (0.11, 0.69)
Household residency (rural)	1.67** (1.20, 2.34)	1.61** (1.14, 2.26)	1.22 (0.94, 1.59)	1.28** (1.13, 1.46)	1.55* (1.06, 2.27)
Household agro-ecological zone					
Mountain	1.00 (ref)	1.00 (ref)	1.00 (ref)	1.00 (ref)	1.00 (ref)
Hill	0.34** (0.20, 0.58)	0.79 (0.49, 1.25)	1.01 (0.62, 1.67)	1.17 (0.95, 1.43)	1.09 (0.62, 1.93)
* Terai*	0.32** (0.18, 0.57)	1.66 (0.98, 2.82)	1.42 (0.82, 2.46)	0.94 (0.75, 1.18)	0.68 (0.32, 1.44)
Living with mother-in-law	1.00 (0.74, 1.36)	1.16 (0.88, 1.53)	1.14 (0.89, 1.46)	1.08 (0.99, 1.18)	1.05 (0.77, 1.45)
Child age in months	–	–	–	0.96** (0.95, 0.97)	1.00 (0.98, 1.03)
Child sex	–	–	–	0.97 (0.90, 1.04)	0.74* (0.56, 0.97)

*ANC* antenatal care, *PNC* postnatal care, *GMP* growth monitoring and promotion, *HMG* health mothers’ group, *OR* odds ratio, *IRR* incidence rate ratio

*P < 0.05

**P < 0.01

## Discussion

In this study, we examined relationships between maternal decision-making input and their health-seeking behaviors within the first 1000-day period. After adjusting for potentially confounding factors, we found that maternal decision-making input was positively associated with receiving at least 4 ANC during pregnancy, attending HMG meetings and making GMP visits in the 6 months prior to the survey, but not with institutional delivery or PNC. It should be noted that health-seeking behaviors are not simply a function of decision-making; many other factors could also greatly affect the utilization of health services, such as control over resources, distance to health clinics, and the availability and accessibility of quality health services in the local context.

Studies testing the linkage between maternal decision-making and the appropriate use of delivery care, including institutional delivery and skilled birth attendance (SBA), have shown mixed results. In contrast to our finding of non-significant associations, 3 recent studies conducted in Ghana and Ethiopia reported a positive relationship (Darega et al. 2016; Speizer et al. 2014; Tekelab et al. 2015), while existing research suggests that mothers who make decisions alone versus with other household members are less likely to have institutional delivery or access SBA (Anyait et al. 2012; Hagos et al. 2014; Mahato et al. 2017; Wilunda et al. 2015). These discrepancies might be attributable to the variations in the measurement or operationalization of decision-making across studies. In addition, socio-cultural factors might mean that having adequate decision-making input does not necessarily translate into delivery in a health facility. In Nepal, social norms that women and daughters-in-law should be obedient and not demanding mean that sometimes young mothers refuse to access institutional delivery, claiming this as their own decision (Brunson 2010). Also, autonomous mothers may opt for home delivery given traditional understandings of childbirth (Bohren et al. 2014; Brunson 2010). Being pregnant and giving birth are sometimes seen as natural and not requiring biomedical interventions unless life-threating complications occur. Furthermore, labor is not always predictable and obstacles beyond empowerment, including remoteness and poor transportation, may prevent institutional delivery as well.

Our results confirm the positive associations reported in the majority of prior research on maternal decision-making and ANC. However, it is noteworthy that even among studies to date in Nepal, measurement of ANC differs across studies: whether ANC at all, at least 4 ANC, and provided by a skilled provider, for instance (Furuta and Salway 2006; Joshi et al. 2014; Situ and Neupane 2016). The linkage between maternal decision-making and PNC is under-studied. Mistry et al. (2009), analyzing 11,648 mothers sampled from the population-based 1998–1999 India National Family Health Survey, found that maternal decision-making autonomy was positively associated with receiving a PNC checkup within 2 months after delivery. Two cross-sectional studies conducted in Ethiopia also reported similar findings that mothers with the ability to make decisions were more likely to make a PNC visit within the 6 weeks after childbirth (Darega et al. 2016; Tesfahun et al. 2014). Our results of non-significant relationships between maternal decision-making input and PNC might be due to the stringent local protocol of having at least 3 PNC visits in the first week after delivery that we applied to construct this outcome variable.

Our findings also suggested that maternal decision-making input was associated with increased frequency of GMP and HMG attendance, two activities advocated by SII to promote children’s physical growth and nutritional well-being in the postpartum period within the first 1000 days. To date, there is a paucity of research directly examining the use of similar health services after childbirth worldwide.

The multidimensionality of maternal decision-making complicates the comparison among published results. Several studies constructed decision-making measures focusing on specific domains, such as household purchases, or health care (Furuta and Salway 2006; Speizer et al. 2014; Tesfahun et al. 2014), while it is more frequently seen in the literature that individual indicators from various decision domains were aggregated into an index as the estimate of maternal decision-making status (Bloom et al. 2001; Mistry et al. 2009; Pratley 2016; Situ and Neupane 2016; Sripad et al. 2019). These and other variations in the operationalization of decision-making for measurement create often make comparisons of the findings from one to the other impossible. There is still no consistent and internationally recognized standard for the valid measurement of maternal decision-making (Pratley 2016). In this study, we aggregated indicators developed in the WEAI to quantify maternal decision-making input, which reflects the instrumental agency she possessed regarding household decision-making (Malapit et al. 2019). This also helped facilitate the comparison between our results and those of previous studies that used aggregated decision-making measures.

This study is, to our knowledge, the first to assess maternal decision-making input in relation to their utilization of ANC, delivery care, PNC, and other health-seeking behaviors within the 1000-day period. The large sample size (N = 1910) used for analyses ensures statistical power to detect a significant association. This study, however, has a few limitations. First, given the cross-sectional study design, we measured a mother’s participation in household decision-making and her health-seeking behaviors at the same time. This limits our ability to establish temporality and confirm causality. It is unlikely, however, that subjects’ participation in decisions on various household activities would change substantially in a short period of time. Second, our results are open to biases inherent in self-reporting such as recall and response bias. In this case, mothers in the survey reside in SII intervention areas and should be aware of the health services promoted by SII. Thus, we cannot rule out social desirability bias. Also, while survey-based continuous measures of household decision-making input are widely used, validation of these measures has been limited (Yount et al. 2019). Given the 2016 recommendation from WHO of 8 ANC visits during pregnancy, Nepal and other countries may slowly transition to that in their national policy and in turn, future related research could seek to improve our understanding predictors of a household receiving the increased number of ANC visits.

## Conclusions

Our findings suggest that maternal decision-making input might play an important role in promoting health-seeking behaviors during the 1000-day period, highlighting the importance of mothers’ active participation in household decision-making activities. While other factors clearly also play a major, and perhaps even larger deterministic role, it is also important to consider maternal decision-making in the planning and implementation of policies and programs in Nepal and other LMICs to make further progress in reducing poor maternal and child health and nutrition. Additional research is needed to supplement the predominance of cross-sectional studies to date; tracking maternal decision-making input longitudinally and throughout first 1000 days would enable further assessment of these relationships.
